# Consensus Tracking for Multiagent Systems with Nonlinear Dynamics

**DOI:** 10.1155/2014/130643

**Published:** 2014-08-14

**Authors:** Runsha Dong

**Affiliations:** The State Key Laboratory for Turbulence and Complex System, Department of Mechanics and Engineering Science, College of Engineering, Peking University, Beijing 100871, China

## Abstract

This paper concerns the problem of consensus tracking for multiagent systems with a dynamical leader. In particular, it proposes the corresponding explicit control laws for multiple first-order nonlinear systems, second-order nonlinear systems, and quite general nonlinear systems based on the leader-follower and the tree shaped network topologies. Several numerical simulations are given to verify the theoretical results.

## 1. Introduction

There have been a lot of recent researches paying attention to the problem of multiagent cooperative control which means a group of agents working cooperatively to achieve coverage, formation, and consensus [1–5]. The consensus problem, known as agreement on certain quantities of interest for groups of agents, is one of the major research directions. Consensus tracking means consensus with a dynamical leader [6, 7]. It is considered as a manner of cooperative behaviors and has also drawn far more attention.

In the pioneering work on consensus tracking of Ren [8], consensus with a constant reference state and with a time-varying reference state is analyzed for the first-order integrator systems. However, even in the second case, the dynamics of the time-varying reference state is assumed to have no explicit external input. Later, many variants of the consensus tracking algorithms are developed for various system models. In [9], a control algorithm is proposed for the problems of consensus tracking while those homogenous follower agents are with dynamics of first-order linear integrator and the leader is governed by the different dynamics. In [10], a consensus tracking algorithm is proposed and analyzed for the second-order integrator dynamics which is also a linear system model. And in [6], the author designs several consensus tracking algorithms for the agents with first-order (and second-order) integrator dynamics. We note that both the follower agents and the leader are with linear dynamics, and the leader has an upper bounded external input.

Li et al. [11] propose an observer-based algorithm for the problem of consensus tracking for multiagent systems with general linear dynamics. In the recent literature [12], the problem of multiple first-order nonlinear systems tracking several leaders is studied under the assumption that these leaders have no explicit external input. In [13], the consensus tracking problem is studied for the case that the dynamics of both the leader and the followers are of the second-order nonlinearity, under the assumption that the leader has no external input.

Nonlinear dynamics are now studied in the consensus problem from various perspectives such as [14–17]. In [16, 17], the effective consensus tracking laws are developed for multiagent systems modeled as higher-order dynamics with nonlinear terms under switching directed topologies. By contrast, this paper discusses the consensus tracking problem for multiagent systems with general nonlinear systems and the very special cases (the first-order and the second-order nonlinear systems) under tree topologies. The network of groups of nonlinear systems is a kind of coupled nonlinear systems with linear coupling (linearly coupled ordinary differential equations) [18] which is widely used in nature and engineering to describe the models of spike-burst neural activity, the transitions of *n*-patch metapopulation, the dynamics of linearly coupled Chua circuits [13, 18], the coupled oscillator systems [19], epidemiology, ecology [20], and so on.

In those papers mentioned above, some focus on the problem that the linear follower agents track a leader who is governed by an external input, yet others focus on the problem that the nonlinear follower agents track a leader who has no explicit external input. In the practical network with a linear or a nonlinear leader, the external input is unavoidable or even is important for guiding the group to behave correctly. Thus, the study of consensus tracking for a group of nonlinear agents with the leader having an external input will be significative. In this paper, we consider the problem of consensus tracking for the network of a group of *N* + 1 identical nonlinear agents, in which one agent indexed by *r* and governed by its external input is assigned to be the leader, and the other agents indexed by 1,…, *N* are regarded as the followers. The nonlinear dynamics of agents in this paper are described by the first-order (resp., the second-order and the general) nonlinear equations like (1) in [12] (resp., (3) in [21] and (1) in [22]) which will be introduced later.

We have noted that the intrinsic dynamics of the leader in [12] which is specified by ${\dot{x}}_{r}=f(t,x_{r})$ have no explicit external input, where *x*_*r*_ is the state of the leader, *f* is the nonlinear vector field, and *t* is the time. However, it can be interpreted as the fact that each follower has known the detailed measurements of the leader's external input *u*(*t*) all the time and the consensus algorithm for the follower could cancel out the impact of the leader's external input. Though the equation *f*(*t*, *x*_*r*_) is theoretically capable of including the situation of *f*(*t*, *x*_*r*_) + *u*(*t*), the given Lipschitz condition for the *f* in [12] will limit the choice of the control input *u*(*t*) or sometimes there will even be no choice. However, in this paper, we relax this condition and assume that each follower only knows the upper bound of the leader's input in advance and there are no other limitations. There is a similar situation in [21].

Due to the existence of nonlinearity in the agents' dynamics and the external input of the leader, the existing consensus algorithms are not applicable to our problem. By synthetically using the Lipschitz conditions, the variable structure technique [6], the feedback linearization technique [22], and the Lyapunov theory, all three control algorithms for consensus tracking under the undirect or the tree shaped communication topology are effectively designed.

The remainder of the paper is organized as follows. In Section 2, some notations and basic concepts in graph theory that will be used in this paper are introduced.  Section 3 is the main text that establishes the consensus tracking algorithms for nonlinear systems.  Section 4 shows several simulation results. Finaly, Section 5 draws conclusions to this paper.

## 2. Background and Preliminaries

We use ||·|| to denote the Euclidean norm and ||·||_1_ 1-norm. Let 1^*n*^, 0^*n*^ denote *n* × 1 column vectors with all components being ones and zeros, respectively. *I*_*n*_ is used to denote the *n* × *n* identity matrix. And ⊗ stands for the kronecker product. A function *f* is said to be of class *C* ^*k*^ if the derivatives *f*′, *f*′′,…, *f* ^(*k*)^ exist and are continuous. The superscript *T* means the transpose of a matrix. For a matrix *M*, *M* > 0 denotes that *M* is positive definite.

Since graph theory plays an important role in modeling the communication topology of the network of the multiagent systems, some basic concepts in graph theory that will be used in this paper are introduced in the following.

In the problem of nonlinear consensus tracking, a kind of communication topology of *N* follower agents is modeled as an undirected graph *G* = {*V*, *E*, *A*}, where *V* = {1,2,…, *i*,…, *N*} is a set of *N* integers, with the number *i* which means the *i*th vertex representing the *i*th agent, and *E* ⊂ *V* × *V* is an edge set in which each edge is denoted by a pair of vertices (*i*, *j*). In an undirected graph, (*i*, *j*) ∈ *E* is equivalent to (*j*, *i*) ∈ *E*. The set of neighbors of agent *i* is denoted by *N*_*i*_ = {*j* ∈ *V* : (*i*, *j*) ∈ *E*}. *A* = [*a*_*ij*_] ∈ *R* ^*N*×*N*^ is a weighted adjacency matrix of *G*, where *a*_*ii*_ = 0 and *a*_*ij*_ = 1 if (*i*, *j*) ∈ *V* or 0 otherwise. The Laplacian of *G* is defined as *L* = *D* − *A*, where *D* = diag⁡(deg_1_,…, deg_*N*_) and deg_*i*_ = ∑_*j*=1_^*N*^ *a*_*ij*_ [23]. A path in an undirected graph *G* is a sequence of edges in the form of  (*i*_1_, *i*_2_), (*i*_2_, *i*_3_),…, where *i*_*k*_ ∈ *V*. An undirected graph is connected if there exists a path between every two vertices.

For a directed graph, (*i*, *j*) ∈ *E* does not necessarily mean (*j*, *i*) ∈ *E*. A directed path is a sequence of directed edges in the form of (*i*_1_, *i*_2_), (*i*_2_, *i*_3_),…, where *i*_*k*_ ∈ *V*. The tree shaped communication topology is modeled as the tree shaped graph (a directed graph) in which each vertex has only one parent vertex except for one vertex called the root. To study the problem of nonlinear consensus tracking, a leader adjacency matrix *H* is defined as *H* = diag⁡(*h*_1_, *h*_2_,…, *h*_*i*_,…, *h*_*N*_), where *h*_*i*_ = 1 if the leader's information is available to the *i*th follower agent and *h*_*i*_ = 0 otherwise. The undirect graph *G* with one additional vertex representing a leader is used to model the leader-follower communication topologies in this paper.

## 3. Nonlinear Consensus Tracking

### 3.1. Consensus Tracking for the First-Order Nonlinear Dynamics

We start by considering the first-order nonlinearity case: *N* followers labeled as 1,2,…, *N* are described by the following first-order nonlinear ordinary differential equation:

(1) $$\begin{array}{c} {\dot{x}}_{i}=f\left(t,x_{i}\right)+u_{i},\;i=1,\ldots ,N, \end{array}$$

where *x*_*i*_ ∈ *R* ^*n*^ is the state vector representing the position of agent *i*, *f* : *R* ^*n*^ × *R* → *R* ^*n*^ is a uniformly continuously differentiable vector-valued function, and *u*_*i*_ ∈ *R* ^*n*^ is the control input. The communication topology of these *N* followers is modeled as an undirected graph *G* = {*V*, *E*, *A*}. The corresponding Laplacian matrix and adjacency matrix are denoted by *L* and *A*. We aim to design a control algorithm *u*_*i*_, *i* = 1,…, *N*, such that

(2) $$\begin{array}{c} \underset{t\to \infty }{\lim}||x_{i}\left(t\right)-x_{r}\left(t\right)||=0, \end{array}$$

where *x*_*r*_ ∈ *R* ^*n*^ is the state vector representing the position of the leader which is specified by

(3) $$\begin{array}{c} {\dot{x}}_{r}=f\left(t,x_{r}\right)+u_{r}. \end{array}$$

Note that *u*_*r*_ is the external input of the leader and *u*_*r*_ ≠ 0^*n*^. If the limit (2) is finally achieved, then we say that the first-order nonlinear followers (1) with the control algorithm asymptotically track the leader (3).

As in most existing works on networks of nonlinear agents [12, 13, 21, 24], we give an assumption of Lipschitz-like condition as follows.

**Assumption 1 .**

There exists *ρ* > 0 such that the vector field *f* : *R* ^*n*^ × *R* → *R* ^*n*^ satisfies ||*f*(*t*, *p*) − *f*(*t*, *q*)|| ≤ *ρ*||*p* − *q*||, for all *p*, *q* ∈ *R* ^*n*^.

In order to guarantee these *N* followers could track the leader, the necessary connectivity is required from the point of view of graph theory. For this, we further make the following assumption.

**Assumption 2 .**

The undirected graph *G* which models the network topology of *N* followers is connected and at least one follower is informed about the state of the leader.

To deal with the problem of consensus tracking for the network with the first-order nonlinear agents, we propose a control algorithm for (1) as

(4) ui=−αi(t)[∑j∈Ni(xi−xj)+hi(xi−xr)] −ωsgn⁡[∑j∈Ni(xi−xj)+hi(xi−xr)],

where *α*_*i*_(*t*) is the adaptive gain [13] for agent *i* and it is specified by

(5) α˙i(t)=βi[∑j∈Ni(xi−xj)+hi(xi−xr)]T ×[∑j∈Ni(xi−xj)+hi(xi−xr)],

where sgn⁡(·) is the signum function, *ω* > ||*u*_*r*_||, *β*_*i*_ is any positive constant, and *h*_*i*_ is used for describing whether agent *i* is informed about the state of the leader, as we introduced in Section 2, and we denote that *H* = diag⁡(*h*_1_, *h*_2_,…, *h*_*i*_,…, *h*_*N*_). The column stack vectors of *x*_*i*_  (*i* ∈ *V*) and *f*(*t*, *x*_*i*_)  (*i* ∈ *V*) are denoted by *x* and *F*(*t*, *x*), respectively. By applying the control algorithms (4) and (5) into the input of the system (1), the closed-loop system is then rewritten as follows:

(6) x˙=−(α(t)⊗In)[(L⊗In)x+(H⊗In)(x−(1N⊗In)xr)] −ωsgn⁡[(L⊗In)x+(H⊗In)(x−(1N⊗In)xr)] +F(t,x),

where *α*(*t*)≜diag⁡(*α*_1_(*t*), *α*_2_(*t*),…, *α*_*N*_(*t*)).

Then, the main result on the problem of consensus tracking for the network with first-order nonlinear agents is proposed by the following theorem.

**Theorem 3 .**

If Assumptions 1 and 2 are satisfied, then the first-order nonlinear followers (1) with the control algorithms (4) and (5) asymptotically track the leader (3).

**Proof**

Let ${\tilde{x}}_{i}=x_{i}-x_{r}$ and $\tilde{x}≜{\left[{\tilde{x}}_{1},{\tilde{x}}_{2},\ldots ,{\tilde{x}}_{N}\right]}^{T}$. Then, we have

(7) x~˙=F(t,x)−1N⊗f(t,xr)−(α(t)⊗In)[(L+H)⊗In]x~ −ωsgn⁡([(L+H)⊗In]x~)−(1N⊗In)ur.

From Assumption 2 and Lemma  1 in [13], the matrix *M*≜*L* + *H* is positive definite. Consider a Lyapunov function candidate

(8) $$\begin{array}{c} V=\frac{1}{2}{\tilde{x}}^{T}\left(M⊗I_{n}\right)\tilde{x}+\overset{N}{\underset{i=1}{\sum }}\frac{1}{2\beta_{i}}{\left(\alpha_{i}\left(t\right)-\alpha_{0}\right)}^{2}, \end{array}$$

where *α*_0_ is chosen such that

(9) $$\begin{array}{c} \alpha_{0}\geq \frac{\rho \lambda_{\max}\left(M\right)}{\lambda_{\min}{\left(M\right)}^{2}}. \end{array}$$

The derivative of *V* along the system (7) satisfies

(10) V˙(t)=x~T(M⊗In)x~˙+1βi∑i=1n(αi(t)−α0)α˙i(t)=x~T(M⊗In)[F(t,x)−1N⊗f(t,xr)] −[(M⊗In)x~]T(α(t)⊗In)(M⊗In)x~ −ω[(M⊗In)x~]T[sgn⁡((M⊗In)x~)+(1N⊗In)ur] +[(M⊗In)x~]T(α(t)⊗In)[(M⊗In)x~] −α0[(M⊗In)x~]T[(M⊗In)x~]≤ρλmax⁡(M)||x~||2−(ω−||ur||)||(M⊗In)x~||1 −α0λmin⁡(M)2||x~||2.

From (9) and *ω* > ||*u*_*r*_||, it is easy to obtain that $\dot{V}<0$. Therefore, $\tilde{x}\to 0$ as *t* → *∞*. It follows that lim⁡_*t*→*∞*_||*x*_*i*_(*t*) − *x*_*r*_(*t*)|| = 0. That is, the first-order nonlinear followers (1) track the leader (3) asymptotically.

### 3.2. Consensus Tracking for the Second-Order Nonlinear Dynamics

Next, we discuss the second-order nonlinearity case. Suppose that each of the *N* followers is described by

(11) $$\begin{array}{c} {\dot{x}}_{i}=v_{i},\\ {\dot{v}}_{i}=f\left(t,x_{i},v_{i}\right)+u_{i}, \end{array}$$

where, *i* = 1,…, *N*, *x*_*i*_ ∈ *R* and *v*_*i*_ ∈ *R* are the state representing the position and the velocity of agent *i*, respectively. *f* : *R* × *R* × *R* → *R* is the intrinsic dynamics. *u*_*i*_ ∈ *R* is the control input. The problem is to design *u*_*i*_ for each of the *N* followers to track the leader which is specified by

(12) $$\begin{array}{c} {\dot{x}}_{r}=v_{r},\\ {\dot{v}}_{r}=f\left(t,x_{r},v_{r}\right)+u_{r}, \end{array}$$

such that, for each agent *i*,

(13) $$\begin{array}{c} \underset{t\to \infty }{\lim}||x_{i}\left(t\right)-x_{r}\left(t\right)||=0,\\ \underset{t\to \infty }{\lim}||v_{i}\left(t\right)-v_{r}\left(t\right)||=0, \end{array}$$

where *x*_*r*_ ∈ *R* and *v*_*r*_ ∈ *R* are, respectively, the position and velocity of the leader. If limits (13) are finally achieved, then we say that the second-order nonlinear followers (11) with the designed control algorithm asymptotically track the leader (12). Before studying this problem, we give some assumptions.

**Assumption 4 .**

There exist *ρ*_1_ > 0 and *ρ*_2_ > 0 such that the vector field *f* : *R* × *R* × *R* → *R* satisfies ||*f*(*t*, *x*, *y*) − *f*(*t*, *z*, *w*)|| ≤ *ρ*_1_||*x* − *z*|| + *ρ*_2_||*y* − *w*||, for all *x*, *y*, *z*, *w* ∈ *R*.

**Remark 5 .**

Compared with Assumption 1, it is easy to see that both of the assumptions are Lipschitz-like conditions.

Similar to the first-order case in Section 3.1, we give the same assumption on communication topology of the network as Assumption 2 to guarantee these *N* followers with dynamics of (11) could track the leader with dynamics of (12). And *M* = *L* + *H* here also represents the communication topology of the network, where *L* and *H* are the same as in Section 3.1.

Then, we propose the following control algorithm applied for the system (11):

(14) ui=−∑j=1Naij[(xi−xj)+α(vi−vj)] −hi[(xi−xr)+α(vi−vr)] −wsgn⁡{∑j=1Naij[γ(xi−xj)+(vi−vj)]+hi[γ(xi−xr)+(vi−vr)]},

where *α* > 0 is a constant gain, *γ* is a positive constant which is to be designed, and *ω* is a constant that satisfies *ω* > ||*u*_*r*_||. Before going any further, we define two matrices *P*_*γ*_ and *Q*_*γ*_ associated with *γ*. For a matrix *M* (let *λ*_*M*_ be the maximal eigenvalue of the *M*) and the constants *α* > 0, *ρ*_1_ > 0 and *ρ*_2_ > 0, *P*_*γ*_, and *Q*_*γ*_ are defined by

(15) $$\begin{array}{c} P_{\gamma }≜\left[\begin{array}{cc} \frac{1}{2}\left(M^{2}-\gamma \lambda_{M}I-\lambda_{M}\rho_{1}\rho_{2}I-\alpha \gamma M^{2}\right) & \frac{\gamma }{2}M\\ \frac{\gamma }{2}M & \frac{1}{2}M \end{array}\right],\\ Q_{\gamma }≜\left[\begin{array}{cc} \gamma M^{2}-\frac{\lambda_{M}}{2}\gamma^{2}I-\frac{\lambda_{M}}{2}{\rho_{1}}^{2}I & 0_{N\times N}\\ 0_{N\times N} & \begin{array}{c} \alpha M^{2}-\gamma M-\frac{\lambda_{M}}{2}I-\frac{\lambda_{M}}{2}{\rho_{2}}^{2}I \end{array} \end{array}\right]. \end{array}$$

Then, we have the following lemma.

**Lemma 6 .**

Given the matrix *M* and the constants *ρ*_1_ > 0, *ρ*_2_ > 0, for any constant *α* > 0, if *γ* satisfies

(16) $$\begin{array}{c} \gamma \in \left\{\gamma \;|\;\max\left(0,c_{4}\right)<\gamma <\min\left\{c_{1},c_{2},c_{3},c_{5},c_{6}\right\}\right\} \end{array}$$

or

(17) $$\begin{array}{c} \gamma \in \left\{\gamma \;|\;\max\left(0,c_{4},c_{5},c_{6}\right)<\gamma <\min\left\{c_{1},c_{2},c_{3}\right\}\right\}, \end{array}$$

then *P*_*γ*_ = *P*_*γ*_^*T*^ > 0 and *Q*_*γ*_ = *Q*_*γ*_^*T*^ > 0, where

(18) $$\begin{array}{c} c_{1}≜\frac{1+\lambda_{M}-\rho_{1}\rho_{2}}{1+\alpha \lambda_{M}},\\ c_{2}≜\sqrt{\frac{{\left(1+\alpha \lambda_{M}\right)}^{2}}{4}+\lambda_{M}-\rho_{1}\rho_{2}}-\frac{\left(1+\alpha \lambda_{M}\right)}{2},\\ c_{3}≜\sqrt{\lambda_{M}^{2}+2\left(\alpha -1\right)\lambda_{M}-\left(\rho_{1}^{2}+\rho_{2}^{2}\right)}+\left(\lambda_{M}-1\right),\\ c_{4}≜\lambda_{M}-\sqrt{\lambda_{M}^{2}-\rho_{1}^{2}},\\ c_{5}≜\lambda_{M}+\sqrt{\lambda_{M}^{2}-\rho_{1}^{2}},\\ c_{6}≜\alpha \lambda_{M}-\frac{1}{2}\left(1+\rho_{1}^{2}\right). \end{array}$$

**Proof**

Since *M* is a positive definite matrix, it can be diagonalized as *M* = Γ^−1^ΛΓ, where Λ = diag⁡(*λ*_1_, *λ*_2_,…, *λ*_*N*_) and *λ*_1_ ≥ *λ*_2_ ≥ ⋯≥*λ*_*N*_. We define that

(19) $$\begin{array}{c} \tilde{\Gamma }=\left[\begin{array}{cc} \Gamma & 0_{N\times N}\\ 0_{N\times N} & \Gamma \end{array}\right]. \end{array}$$

It then follows that

(20) $$\begin{array}{c} P_{\gamma }={\tilde{\Gamma }}^{-1}\underset{{\tilde{P}}_{\gamma }}{\underset{︸}{\left[\begin{array}{cc} \frac{1}{2}\left(\Lambda^{2}-\gamma \lambda_{M}I-\lambda_{M}\rho_{1}\rho_{2}I-\alpha \gamma \Lambda^{2}\right) & \frac{\gamma }{2}\Lambda \\ \frac{\gamma }{2}\Lambda & \frac{1}{2}\Lambda \end{array}\right]}}\tilde{\Gamma }. \end{array}$$

Let *η* be any eigenvalue of the matrix ${\tilde{P}}_{\gamma }$. Since Λ is a diagonal matrix and ${\tilde{P}}_{\gamma }$ is symmetric, it follows that *η* is real and satisfies

(21) [η−12(λi2−γλM−λMρ1ρ2−αγλi2)][η−12λi] −γ24λi2=0.

That is,

(22) η2−[12(λi2−γλM−λMρ1ρ2−αγλi2)+12λi]η +14λi(λi2−γλM−λMρ1ρ2−αγλi2)−γ24λi2=0.

Note that *η* > 0 if and only if

(23) $$\begin{array}{c} \frac{1}{2}\left(\lambda_{i}^{2}-\gamma \lambda_{M}-\lambda_{M}\rho_{1}\rho_{2}-\alpha \gamma \lambda_{i}^{2}\right)+\frac{1}{2}\lambda_{i}>0,\\ \frac{1}{4}\lambda_{i}\left(\lambda_{i}^{2}-\gamma \lambda_{M}-\lambda_{M}\rho_{1}\rho_{2}-\alpha \gamma \lambda_{i}^{2}\right)-\frac{\gamma^{2}}{4}\lambda_{i}^{2}>0, \end{array}$$

which means

(24) $$\begin{array}{c} \gamma <\frac{1+\lambda_{M}-\rho_{1}\rho_{2}}{1+\alpha \lambda_{M}}≜c_{1},\\ \gamma <\sqrt{\frac{{\left(1+\alpha \lambda_{M}\right)}^{2}}{4}+\lambda_{M}-\rho_{1}\rho_{2}}-\frac{\left(1+\alpha \lambda_{M}\right)}{2}≜c_{2}. \end{array}$$

By a similar analysis, we have

(25) $$\begin{array}{c} Q_{\gamma }={\tilde{\Gamma }}^{-1}\underset{{\tilde{Q}}_{\gamma }}{\underset{︸}{\left[\begin{array}{cc} \gamma \Lambda^{2}-\frac{\lambda_{M}}{2}\gamma^{2}-\frac{\lambda_{M}}{2}\rho_{1}^{2} & 0_{N\times N}\\ 0_{N\times N} & \begin{array}{c} \alpha \Lambda^{2}-\gamma \Lambda -\frac{\lambda_{M}}{2}-\frac{\lambda_{M}}{2}\rho_{2}^{2} \end{array} \end{array}\right]}}\tilde{\Gamma }. \end{array}$$

Let *μ* be any eigenvalue of the matrix ${\tilde{Q}}_{\gamma }$. Then, one has

(26) [μ−(γλi2−λM2γ2−λM2ρ12)] ×[μ−(αλi2−γλi−λM2−λM2ρ22)]=0.

And *μ* > 0 if and only if

(27) $$\begin{array}{c} \gamma \lambda_{i}^{2}-\frac{\lambda_{M}}{2}\gamma^{2}-\frac{\lambda_{M}}{2}\rho_{1}^{2}+\alpha \lambda_{i}^{2}-\gamma \lambda_{i}-\frac{\lambda_{M}}{2}-\frac{\lambda_{M}}{2}\rho_{2}^{2}>0,\\ \left(\gamma \lambda_{i}^{2}-\frac{\lambda_{M}}{2}\gamma^{2}-\frac{\lambda_{M}}{2}\rho_{1}^{2}\right)\left(\alpha \lambda_{i}^{2}-\gamma \lambda_{i}-\frac{\lambda_{M}}{2}-\frac{\lambda_{M}}{2}\rho_{2}^{2}\right)>0, \end{array}$$

which means

(28) $$\begin{array}{c} \gamma <\sqrt{\lambda_{M}^{2}+2\left(\alpha -1\right)\lambda_{M}-\left(\rho_{1}^{2}+\rho_{2}^{2}\right)}+\left(\lambda_{M}-1\right)≜c_{3},\\ c_{4}≜\lambda_{M}-\sqrt{\lambda_{M}^{2}-\rho_{1}^{2}}<\gamma <\lambda_{M}+\sqrt{\lambda_{M}^{2}-\rho_{1}^{2}}≜c_{5},\\ \gamma <\alpha \lambda_{M}-\frac{1}{2}\left(1+\rho_{1}^{2}\right)≜c_{6}, \end{array}$$

or

(29) $$\begin{array}{c} \gamma <\sqrt{\lambda_{M}^{2}+2\left(\alpha -1\right)\lambda_{M}-\left(\rho_{1}^{2}+\rho_{2}^{2}\right)}+\left(\lambda_{M}-1\right)≜c_{3},\\ \gamma >\lambda_{M}+\sqrt{\lambda_{M}^{2}-\rho_{1}^{2}}≜c_{5},\\ \gamma >\alpha \lambda_{M}-\frac{1}{2}\left(1+\rho_{1}^{2}\right)≜c_{6}. \end{array}$$

In summary, if *γ* satisfies *γ* ∈ {*γ*∣max⁡(0, *c*_4_) < *γ* < min⁡{*c*_1_, *c*_2_, *c*_3_, *c*_5_, *c*_6_}} or *γ* ∈ {*γ*∣max⁡(0, *c*_5_, *c*_6_) < *γ* < min⁡{*c*_1_, *c*_2_, *c*_3_}}, then both the matrix ${\tilde{P}}_{\gamma }$ and the matrix ${\tilde{Q}}_{\gamma }$ are positive definite. Since ${\tilde{P}}_{\gamma }$ and ${\tilde{Q}}_{\gamma }$ have the same eigenvalues as that of *P*_*γ*_ and *Q*_*γ*_, we have *P*_*γ*_ = *P*_*γ*_^*T*^ > 0 and *Q*_*γ*_ = *Q*_*γ*_^*T*^ > 0 when *γ* satisfies *γ* ∈ {*γ*∣max⁡(0, *c*_4_) < *γ* < min⁡{*c*_1_, *c*_2_, *c*_3_, *c*_5_, *c*_6_}} or *γ* ∈ {*γ*∣max⁡(0, *c*_5_, *c*_6_) < *γ* < min⁡{*c*_1_, *c*_2_, *c*_3_}}.

Then, the main result follows.

**Theorem 7 .**

Suppose that Assumptions 2 and 4 are satisfied; if *α* > 0 and *γ* ∈ {max⁡{0, *c*_4_} < *γ* < min⁡{*c*_1_, *c*_2_, *c*_3_, *c*_5_, *c*_6_}} ∪ {max⁡(0, *c*_5_, *c*_6_) < *γ* < min⁡{*c*_1_, *c*_2_, *c*_3_}}, where *c*_*i*_,  *i* = 1,2,…, 6, are defined as in Lemma 6, then the second-order nonlinear followers (11) with the control algorithms (14) asymptotically track the leader (12).

**Proof**

Let ${\tilde{x}}_{i}=x_{i}-x_{r}$ and ${\tilde{v}}_{i}=v_{i}-v_{r}$. And let $\tilde{x}={\left[{\tilde{x}}_{1},{\tilde{x}}_{2},\ldots ,{\tilde{x}}_{N}\right]}^{T}$, $\tilde{v}={\left[{\tilde{v}}_{1},{\tilde{v}}_{2},\ldots ,{\tilde{v}}_{N}\right]}^{T}$. We rewrite the closed-loop system of (11) using the control algorithm (14) as

(30) x~˙=v~,v~˙=F(t,x,v)−1N⊗f(t,xr,vr)−Mx~ −αMv~−βsgn⁡{M(γx~+v~)}−1N⊗ur,

where

(31) F(t,x,v) =[f(t,x1,v1),f(t,x2,v2),…,f(t,xN,vN)]T.

Consider a Lyapunov function candidate

(32) V=[x~Tv~T]Pγ[x~v~]=12x~T(M2−γλMI−λMρ1ρ2I−αγM2)x~ +x~TγMv~+12v~TMv~.

From Lemma 6, one has *V* > 0. The derivative of *V* along the system (30) is

(33) V˙=x~T(M2−γλMI−λMρ1ρ2I−αγM2)v~ +v~TγMv~T+x~TγMv~˙+v~TMv~˙=x~T(M2−γλMI−λMρ1ρ2I−αγM2)v~ +v~TγMv~T+(γx~T+v~T)M ×{F(t,x,v)−1N⊗f(t,xr,vr)} −(γx~T+v~T)M(Mx~+αMv~) −(γx~T+v~T)M{βsgn⁡[M(γx~+v~)]+1N⊗ur}.

For the vectors *x*, *y* ∈ *R* ^*N*^ and the matrix 0 < *M* = *M* ^*T*^ ∈ *R* ^*N*×*N*^, we define

(34) $$\begin{array}{c} \langle x,y\rangle =x^{T}My,\;\;{||x||}_{M}={\langle x,x\rangle }^{1/2}={\left(x^{T}Mx\right)}^{1/2}. \end{array}$$

From Cauchy-Schwarz inequality, one has

(35) $$\begin{array}{c} {||xy||}_{M}^{2}\leq {||x||}_{M}{||y||}_{M},\\ x^{T}My\leq {\left(x^{T}Mx\right)}^{1/2}\times {\left(y^{T}My\right)}^{1/2},\;\;{\left(ab\right)}^{1/2}\leq \frac{1}{2}\left(a+b\right). \end{array}$$

It follows that

(36) (γx~T+v~T)M{F(t,x,v)−1N⊗f(t,xr,vr)}≤(γx~T+v~T)M(γx~+v~)(ρ1x~T+ρ2v~T)M(ρ1x~+ρ2v~)≤λM2 ×(γ2x~Tx~+2γx~Tv~+v~Tv~+ρ12x~Tx~+2ρ1ρ2x~Tv~+ρ22v~Tv~)=λMγ22x~Tx~+λMγx~Tv~+λM2v~Tv~ +λM2ρ12x~Tx~+λMρ1ρ2x~Tv~+λM2ρ22v~Tv~,V˙≤x~T(M2−γλMI−λMρ1ρ2I−αγM2)v~ +v~TγMv~T−(γx~T+v~T)M2(x~+αv~) +λMγ22x~Tx~+λMγx~Tv~+λM2v~Tv~+λM2ρ12x~Tx~ +λMρ1ρ2x~Tv~+λM2ρ22v~Tv~ −(γx~T+v~T)M{βsgn⁡{M(γx~+v~)}+1N⊗ur}=x~TM2v~−γλMx~Tv~−λMρ1ρ2x~Tv~−αγx~TM2v~ +v~TγMv~T−γx~TM2x~−x~TM2v~−γαx~TM2v~ −αv~TM2v~+λMγ22x~Tx~+λMγx~Tv~+λM2v~Tv~ +λM2ρ12x~Tx~+λMρ1ρ2x~Tv~+λM2ρ22v~Tv~ −(β−||ur||)||M(γx~+v~)||1=−[x~Tv~T]Qγ[x~v~]−(β−||ur||)||M(γx~+v~)||1.

From Lemma 6 that *Q*_*γ*_ > 0 and *β* − ||*u*_*r*_|| > 0, we have $\dot{V}<0$. Equivalently, it follows that as *t* → *∞*, ${\tilde{x}}_{i}\to 0$, ${\tilde{v}}_{i}\to 0$, which means *x*_*i*_ → *x*_*r*_, *v*_*i*_ → *v*_*r*_ as *t* → *∞*. And then the second-order nonlinear followers (11) with the control algorithms (14) asymptotically track the leader (12).

**Remark 8 .**

In order to deal with the nonlinear term of the agents' dynamics in Theorem 7, one key procedure is the definition of the inner product (34) and the application of Cauchy-Schwarz inequality.

**Remark 9 .**

The result in Theorem 7 for multiagent systems with *x*_*i*_ ∈ *R* and *v*_*i*_ ∈ *R* is also suitable for agents with dynamics evolved in higher-order dimension; that is, *x*_*i*_ ∈ *R* ^*n*^ and *v*_*i*_ ∈ *R* ^*n*^.

### 3.3. Consensus Tracking for the General Nonlinear Dynamics

In the general nonlinear case, suppose that a network system with *N* followers represented by the following nonlinear equation:

(37) $$\begin{array}{c} {\dot{x}}_{i}=f\left(x_{i},u_{i}\right),\;i=1,2,\ldots ,N, \end{array}$$

where *x*_*i*_ ∈ *R* ^*n*^ is the state vector of the *i*th follower and *u*_*i*_ ∈ *R* ^*p*^ is the control input. And a leader is given by

(38) $$\begin{array}{c} {\dot{x}}_{r}=f\left(x_{r},u_{r}\right), \end{array}$$

where *x*_*r*_ ∈ *R* ^*n*^ and *u*_*r*_ ∈ *R* ^*p*^ are, respectively, the state and the control input of the leader. The function *f*(*x*_*i*_, *u*_*i*_) will be *C* ^2^ with regard to *x*_*i*_ and *u*_*i*_, and so is *f*(*x*_*r*_, *u*_*r*_). We aim to give an explicit control law *u*_*i*_ for each follower such that

(39) $$\begin{array}{c} \underset{t\to \infty }{\lim}||x_{i}\left(t\right)-x_{r}\left(t\right)||=0. \end{array}$$

If the limit (39) is finally achieved, then we say that the general nonlinear followers (37) with the control algorithm asymptotically track the leader  (38).

**Remark 10 .**

Note that the differential equation (37) can describe the models of many kinds of mechanical system such as nonholonomic system and underactuated system.

Throughout the subsequent analysis we assume that the network topology satisfies the following two assumptions.

**Assumption 11 .**

The graph of the network topology is tree shaped with the leader as the root node, where the tree shaped graph means each node has only one parent node except the root node.

**Assumption 12 .**

For the network system, each agent knows the measurement of the control input of its parent agent at the same time.

Assumption 12 illustrates that each agent is a cooperative partner with its neighbors. Due to the very general nonlinear dynamics and the goal of nonlinear consensus tracking, it is necessary for an agent to know the input of its parent agent. Motivated by the consensus analysis in [25], where the tool of incidence matrix is used to model the error system, we number the edges in the tree shaped graph according to the length of the path which is indirectly connected to the root node shown in Figure 4.

In order to propose the consensus tracking algorithm for agents (37), we make some preparation. The following analysis is based on the* proposition  1* in [22]. For the tree shaped graph, each follower node *i* in the form of (37) tracks the trajectory of its parent node *j* in the form of (37) (or (38)), where both *x*_*i*_(*t*), *x*_*j*_(*t*) and *u*_*i*_(*t*), *u*_*j*_(*t*) are bounded. And we denote

(40) $$\begin{array}{c} A_{ij}\left(t\right):=\frac{\partial f}{\partial x_{i}}\left(x_{j}\left(t\right),u_{j}\left(t\right)\right),\\ B_{ij}\left(t\right):=\frac{\partial f}{\partial u_{i}}\left(x_{j}\left(t\right),u_{j}\left(t\right)\right). \end{array}$$

Let Φ_*ij*_(*t*, *t*_0_) ∈ *R* ^*n*×*n*^ × *R* be the state transition matrix of *A*_*ij*_(*t*); that is, Φ_*ij*_(*t*, *t*_0_) satisfies ${\dot{\Phi }}_{ij}(t,t_{0})=A_{ij}(t)\Phi_{ij}(t,t_{0})$ with Φ_*ij*_(*t*_0_, *t*_0_) = *I*. Further, for a given constant *α* > 0, we define

(41) Hij(t0,t)=∫t0texp⁡(6α(t0−τ))Φij(t0,τ)Bij(τ)Bij(τ)TΦij(t0,τ)Tdτ.

If there exists a constant *δ* such that *H*_*ij*_(*t*, *t* + *δ*) is bounded away from singularity uniformly in *t*, then define *P*_*ij*_(*t*) as follows:

(42) $$\begin{array}{c} P_{ij}\left(t\right):=H_{ij}^{-1}\left(t,t+\delta \right). \end{array}$$

If there exist two numbers *p*_*ij*_^*m*^ and *p*_*ij*_^*M*^ such that

(43) $$\begin{array}{c} 0<p_{ij}^{m}I<P_{ij}\left(t\right)<p_{ij}^{M}I,\;\forall t\in R_{+}, \end{array}$$

then, for any function *γ*_*ij*_(*t*) : *R*_+_ → [1/2, *∞*), continuous and bounded, we propose the following linear time-varying feedback control law:

(44) $$\begin{array}{c} u_{i}\left(t\right)=u_{j}\left(t\right)-\gamma_{ij}\left(t\right)B_{ij}{\left(t\right)}^{T}P_{ij}\left(t\right)\left[x_{i}\left(t\right)-x_{j}\left(t\right)\right]. \end{array}$$

Now we have the main result as follows.

**Theorem 13 .**

If Assumptions 11 and 12 are satisfied, then the general nonlinear followers (37) with the control algorithms (44) asymptotically track the leader (38).

**Proof**

Let

(45) $$\begin{array}{c} x_{i}\left(t\right)-x_{j}\left(t\right)={\tilde{x}}_{ij}\left(t\right),\\ u_{i}\left(t\right)-u_{j}\left(t\right)={\tilde{u}}_{ij}\left(t\right), \end{array}$$

where *x*_*j*_ and *u*_*j*_ are the state and the input of agent *j* and the agent *j* is the parent agent of agent *i*. For simplicity, we denote ${\tilde{x}}_{ij}(t)$ in the multiagent systems by *e*_*k*_(*t*), *k* = 1,2,…, *m*, like in Figure 4, where *m* is the number of edges in the graph of the network topology, and *e* = [*e*_1_, *e*_2_,…, *e*_*m*_]^*T*^. Similarly, let *P*(*t*) = diag⁡(*P*_1_(*t*), *P*_2_(*t*),…, *P*_*m*_(*t*)), where *P*_*k*_(*t*), *k* = 1,2,…, *m*, represent the matrix *P*_*ij*_(*t*), *i* ∈ *V*,  *j* ∈ *V* ∪ {*r*} (*r* represents the leader in the network), and each is described by (42). Consider a Lyapunov function candidate

(46) $$\begin{array}{c} V=\overset{m}{\underset{k=1}{\sum }}V_{k}=\overset{m}{\underset{k=1}{\sum }}e_{k}^{T}P_{k}e_{k}. \end{array}$$

Note that

(47) u~k(t)=u~ij(t)=−γij(t)Bij(t)TPij(t)x~ij(t)=−γk(t)Bk(t)TPk(t)ek(t).

In addition, one has

(48) e˙k(t)=Ak(t)ek(t)+Bk(t)u~k(t)+o(ek(t),u~k(t),t),P˙k(t)=−Pk(t)H˙k(t)Pk(t),H˙k(t)=H˙ij(t,t+δ)=6αHk(t)+Ak(t)Hk(t)+Hk(t)Ak(t)T +exp⁡(−6αδ)Φk(t,t+δ)Bk(t+δ) ×Bk(t+δ)TΦk(t,t+δ)T−Bk(t)Bk(t)T.

Since *γ*_*k*_(*t*) ≥ 1/2, *k* = 1,2,…, *m*, from the control algorithm (44) and the notation (45) and

(49) $$\begin{array}{c} ||{\tilde{u}}_{k}||=||-\gamma_{k}B_{k}^{T}P_{k}e_{k}||\leq K||e_{k}||,\;K<\infty , \end{array}$$

we have

(50) $$\begin{array}{c} o\left(e_{k}\left(t\right),{\tilde{u}}_{k}\left(t\right),t\right)=\hat{o}\left(e_{k}\left(t\right),t\right),\\ \underset{||e_{k}||\to 0}{\lim}\underset{t\geq 0}{\sup}\frac{||\hat{o}\left(e_{k}\left(t\right),t\right)||}{||e_{k}\left(t\right)||}=0. \end{array}$$

Then,

(51) V˙k(ek,t) =−ek(t)T6αPk(t)ek(t)  −ek(t)T(2γk(t)−1)Pk(t)Bk(t)Bk(t)TPk(t)ek(t)  −ek(t)T[exp⁡(−6αδ)Pk(t)Φk(t,t+δ)Bk(t+δ)×Bk(t+δ)TΦk(t,t+δ)TPk(t)]ek(t)  +2ek(t)TPk(t)o^(ek(t),t) ≤−4αpkm||ek||2.

It follows that $\dot{V}<0$ and ${\tilde{x}}_{ij}\to 0$, ${\tilde{u}}_{ij}\to 0$ as *t* → *∞*. At the same time, lim⁡_*t*→*∞*_||*x*_*i*_(*t*) − *x*_*r*_(*t*)|| = 0 is satisfied and the consensus tracking problem is solved.

**Remark 14 .**

For an undirected connected graph which contains a tree shaped subgraph, or a directed graph which contains a directed spanning tree, we can choose such a tree as Assumption 11 required. However, such a directed tree is unfavorable for implementing the distributed control since in such case each follower has to know the information of the global communication topology. So, it is worth discussing the general undirected (or directed) communication topologies and it will be a direction of the future research.

**Remark 15 .**

It has been shown that systems (1) and (11) are the special cases of system (37). However, the analysis for convergence of closed system is completely different. Though, in each case, the communication topologies are relatively simple.

## 4. Simulation Results

**Figure 1:**
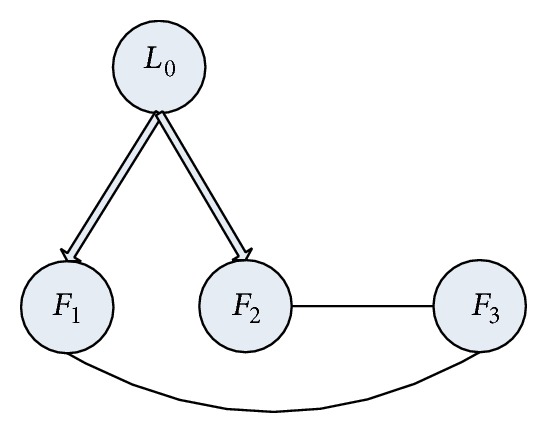
The undirected graph for a group of three followers with a leader. Here *L*_0_ denotes the leader and *F*_*i*_, *i* = 1,2, 3, denote the followers. The direct arrows represent the information flow from the leader to the follower and the indirect edges represent the bidirectional information flow between followers.

In this section, three numerical simulation examples are given to illustrate the theoretical results. Consider the first example, a network of three followers with a leader shown in Figure 1. Assume that the dynamics of the follower agents with *n* = 2 are described by the following equations:

(52) $$\begin{array}{c} f\left(t,x_{i}\right)=\left(\begin{array}{c} x_{i1}\sin t\\ x_{i2}\cos t \end{array}\right),\\ f\left(t,x_{r}\right)=\left(\begin{array}{c} x_{r1}\sin t\\ x_{r2}\cos t \end{array}\right). \end{array}$$

**Figure 2:**
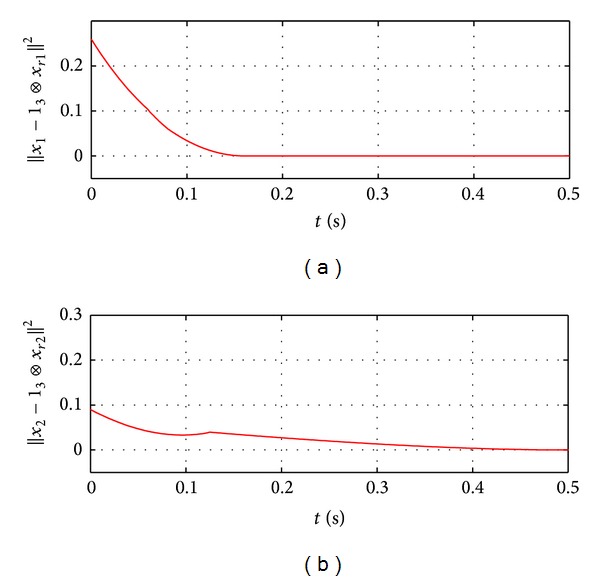
Consensus tracking for the first-order nonlinear systems.

The control input of the leader is given by *u*_*r*_ = [1,1]^*T*^. Choose *β*_*i*_ = 1 for *i* = 1,2, 3 and *ω* = 1.5. Note that $1.5>||u_{r}||=\sqrt{2}$. The initial values of the error between the multiple followers and the leader, which is described by ${\tilde{x}}_{ij}=x_{ij}-x_{rj}$, *i* = 1,2, 3, *j* = 1,2, are given as [0.1, −0.2,0.3, −0.1,0.4,0.2]^*T*^. Then the results of consensus tracking are shown in Figure 2, where *x*_1_ = [*x*_11_, *x*_21_, *x*_31_]^*T*^ and *x*_2_ = [*x*_12_, *x*_22_, *x*_32_]^*T*^. Since ${\tilde{x}}_{ij}\to 0$ as *t* moves on, consensus tracking is finally achieved.

The second example is also given for the graph in Figure 1, which characterizes the communication channel among the three followers and a leader. The dynamics of each follower and the leader are specified by the following equations, respectively:

(53) $$\begin{array}{c} {\dot{x}}_{i}=v_{i},\\ {\dot{v}}_{i}=x_{i}\sin\left(t\right)+v_{i}\cos\left(t\right)+u_{i},\\ {\dot{x}}_{r}=v_{r},\\ {\dot{v}}_{r}=x_{r}\sin\left(t\right)+v_{r}\cos\left(t\right)+u_{r}. \end{array}$$

Note that

(54) $$\begin{array}{c} M=\left[\begin{array}{ccc} 2 & 0 & -1\\ 0 & 2 & -1\\ -1 & -1 & 2 \end{array}\right], \end{array}$$

and *λ*_*M*_ = 3.4142, *ρ*_1_ = *ρ*_2_ = 1. Choose *α* = 1; then it is easy to compute that

(55) $$\begin{array}{c} c_{1}=0.7735,\;\;c_{2}=0.4921,\;\;c_{3}=5.5217,\\ c_{4}=0.1497,\;\;c_{5}=6.6787,\;\;c_{6}=2.4142. \end{array}$$

**Figure 3:**
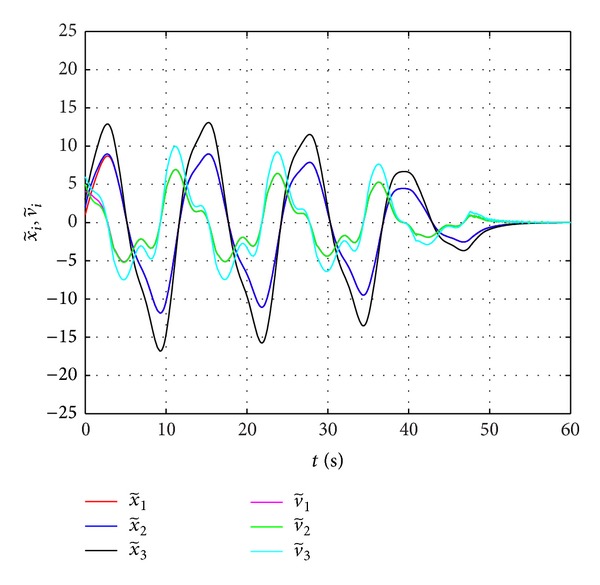
Consensus tracking for the second-order nonlinear systems.

Choose *γ* = 0.45 ∈ {*γ*∣max⁡(0, *c*_4_) < *γ* < min⁡{*c*_1_, *c*_2_, *c*_3_, *c*_5_, *c*_6_}}. Given the initial values as ${\tilde{x}}_{1}(0)=1$, ${\tilde{x}}_{2}(0)=2$, ${\tilde{x}}_{3}(0)=3$, ${\tilde{v}}_{1}(0)=4$, ${\tilde{v}}_{2}(0)=5$, and ${\tilde{v}}_{3}(0)=6$, the results of consensus tracking are shown in Figure 3.

**Figure 4:**
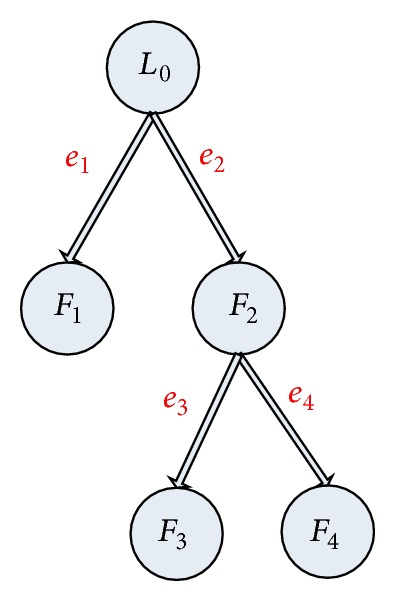
The tree shaped graph for a network of four followers with a leader, where *L*_0_ denotes the leader and *F*_*i*_, *i* = 1,2, 3,4, denote the followers. The direct arrows represent the information flow from the parent agent to the child agent.

Now let us see the third example. In the case of the general nonlinear dynamics, the graph of the network topology is shown in Figure 4.

We consider each agent's dynamics to be a simple nonholonomic system specified by the equations as follows:

(56) $$\begin{array}{c} {\dot{x}}_{1}=u_{1},\\ {\dot{x}}_{2}=u_{2},\\ {\dot{x}}_{3}=x_{2}u_{1}. \end{array}$$

**Figure 5:**
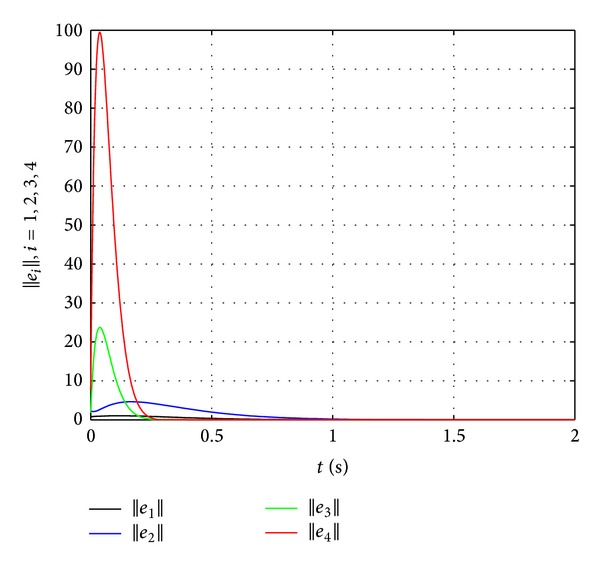
The norm of error *e*_*i*_, *i* = 1,2, 3,4, versus time.

Assume that the trajectory and the control input of the leader are, respectively, described by *x* ^*L*_0_^ = (0, *t*, 0) and *u* ^*L*_0_^ = (0,1), where the superscript *L*_0_ means the leader. Choose *δ* = 0.1 and *γ*_*ij*_(*t*) = 1. For the initial value of *F*_*i*_, *i* = 1,2, 3,4, given by *x* ^*F*_1_^(0) = (0.4, −0.6,0.4), *x* ^*F*_2_^(0) = (−0.8,1.2,1.8), *x* ^*F*_3_^(0) = (0,2.4,1.4), and *x* ^*F*_4_^(0) = (1, −3.8,2.8), the norm results of tracking error *e*_1_(*t*) = *x* ^*F*_1_^ − *x* ^*L*_0_^, *e*_2_(*t*) = *x* ^*F*_2_^ − *x* ^*L*_0_^, *e*_3_(*t*) = *x* ^*F*_3_^ − *x* ^*F*_2_^, and *e*_4_(*t*) = *x* ^*F*_4_^ − *x* ^*F*_2_^ are shown in Figure 5.

Since *e*_*i*_, *i* = 1,2, 3,4, converge to 0 as time moves on, consensus tracking is achieved asymptotically.

## 5. Conclusion

In this paper, we studied the problem of nonlinear consensus tracking via the variable structure technique, the feedback linearization technique, and the Lyapunov theory when there is a leader governed by the external input. Suppose that the leader's external input is upper bounded and a connectivity requirement for the network topology is satisfied; we proposed the consensus tracking algorithms for the followers with the first-order nonlinear dynamics, the second-order nonlinear dynamics, and the general nonlinear dynamics to asymptotically track the corresponding nonlinear leader. And several numerical simulations were given to show the effectiveness of our algorithms. The future works include the study of nonlinear consensus tracking in the general directed network topologies.
